# Cellulose-starch Hybrid Films Plasticized by Aqueous ZnCl_2_ Solution

**DOI:** 10.3390/ijms20030474

**Published:** 2019-01-22

**Authors:** Xiaoqin Shang, Huihua Jiang, Qingling Wang, Peng Liu, Fengwei Xie

**Affiliations:** 1 School of Chemistry and Chemical Engineering, Guangzhou University, Guangzhou 510006, China; hushanren@163.com (X.S.); 15622391828@163.com (H.J.); wql072388@163.com (Q.W.); 2 Fine Chemical Research Institute, Guangzhou University, Guangzhou 510006, China; 3 Institute of Advanced Study, University of Warwick, Coventry CV4 7HS, UK; 4 International Institute for Nanocomposites Manufacturing (IINM), WMG, University of Warwick, Coventry CV4 7AL, UK; 5 School of Chemical Engineering, The University of Queensland, Brisbane, Qld 4072, Australia

**Keywords:** cellulose-starch blend films, ZnCl_2_ solution, rheological properties, mechanical properties, morphology, crystallinity, polysaccharides, natural polymers, solution casting, compression molding

## Abstract

Starch and cellulose are two typical natural polymers from plants that have similar chemical structures. The blending of these two biopolymers for materials development is an interesting topic, although how their molecular interactions could influence the conformation and properties of the resultant materials has not been studied extensively. Herein, the rheological properties of cellulose/starch/ZnCl_2_ solutions were studied, and the structures and properties of cellulose-starch hybrid films were characterized. The rheological study shows that compared with starch (containing mostly amylose), cellulose contributed more to the solution’s viscosity and has a stronger shear-thinning behavior. A comparison between the experimental and calculated zero-shear-rate viscosities indicates that compact complexes (interfacial interactions) formed between cellulose and starch with ≤50 wt % cellulose content, whereas a loose structure (phase separation) existed with ≥70 wt % cellulose content. For starch-rich hybrid films prepared by compression molding, less than 7 wt % of cellulose was found to improve the mechanical properties despite the reduced crystallinity of the starch; for cellulose-rich hybrid films, a higher content of starch reduced the material properties, although the chemical interactions were not apparently influenced. It is concluded that the mechanical properties of biopolymer films were mainly affected by the structural conformation, as indicated by the rheological results.

## 1. Introduction

In recent years, sustainable polymers from renewable resources, which can be used for both high-value areas such as biomedical materials and in basic applications such as packaging, have attracted huge interest [1]. Along with that, various sustainable polymers have attracted huge interest for materials development. These include natural polymers such as cellulose [2,3,4,5,6,7], starch [8,9], chitosan [10,11,12,13,14,15], and alginate [16,17,18,19,20] and biobased polymers such as polylactic acid (PLA) [21,22,23] and polyhydroxyalkanoate (PHA). In particular, starch and cellulose, which are two polysaccharides directly from plants, are abundant in nature and widely available; thus, they can be considered to be promising sources for developing biodegradable materials. 

Starch and cellulose are composed of the same d-glucose unit, referred to as homoglucan or glucopyranose, but linked through different glycosidic bonds. Starch contains two types of biomacromolecules, namely amylose and amylopectin. Amylose is a sparsely branched carbohydrate mainly based on α-1,4 linkages with a molecular weight of 10^5^–10^6^ and has a degree of polymerization (DP) as high as 600 [24]. Amylopectin is also based on α-1,4 linkages (about 95%) but is a multiple-branched polymer via α-1,6 linkages (about 5%), with a high molecular weight of 10^7^–10^9^ [24]. On the other hand, cellulose is a linear polysaccharide, linked through β-1,4 glycosidic bonds with the chain length highly dependent on the origin and treatment of the raw material (e.g., wood pulp, DP = 300) [25]. Microcrystalline cellulose (MCC) is crystalline cellulose derived from high-quality wood pulp, and it is expected to disintegrate into cellulose whiskers after complete hydrolysis [26,27]. MCC has the advantage of a high specific surface area compared with other conventional cellulose fibers [28,29]. Thus, MCC could have more potential than raw cellulose for materials development. 

The development of polymer blends and composites has been considered to be one of the most cost-effective methods of modifying the bulk properties of individual polymers, achieving enhanced and/or new material properties, reducing costs, and expanding the applications of polymeric materials [8,16,30,31,32,33,34,35,36,37]. While cellulose-starch hybrid materials have already been studied widely, most of the work has focused on starch matrices reinforced by cellulose nanowhiskers/nanofibers [38,39,40,41,42,43,44]. Moreover, MCC has been used as the reinforcing agent for starch-based materials, improving the mechanical properties and decreasing the water sensitivity, which can be ascribed to the good interactions between cellulose and starch [45]. Some other studies have investigated materials-based starch and chemically modified cellulose [45,46,47,48,49,50,51], because native cellulose is not processable as a thermoplastic material. However, chemically modified cellulose may present varied properties, which could lead to its phase separation with starch. Limited studies have explored the interaction and compatibility between native starch and native cellulose, especially at a wide range of ratios.

Plasticizers or solvents are very important for the processing of sustainable polymers into bioplastics [52]. Recently, ionic liquids (ILs) have been touted as “green” solvents for natural polymers and have attracted much attention. ILs have the ability to fully or partially disrupt the intermolecular hydrogen bonding present in biopolymeric networks and, as a result, either fully dissolve or plasticize many biopolymers such as starch [2,29,53,54,55,56,57,58,59,60,61,62,63,64,65,66] and cellulose [67,68,69,70,71,72]. For instance, 1-butyl-3-methylimidazolium chloride ([Bmim][OAc]), 1-allyl-3-methylimidazolium chloride ([Amim][Cl]), and 1-ethyl-3-methylimidazolium acetate ([Emin][OAc]) have been applied to the development of cellulose-starch hybrid materials [73,74]. However, the toxicity of ILs has not been fully understood and has been a concern [75], in addition to their high prices. Alternatively, cheap inorganic molten salts have been found to be able to dissolve cellulose as well [76]. Studies have shown that zinc chloride solutions of certain concentrations could dissolve biopolymers such as starch [77,78,79] and cellulose [80,81] efficiently. The treatment of cellulose or starch with zinc inorganic salts has been found to result in the formation of cellulose–zinc complexes [82,83,84] or starch–zinc complexes [85,86]. Recently, starch-based materials plasticized by ZnCl_2_ solutions have been observed to present increased tensile strength by more than threefold at no cost to the elongation at the break [79].

The purpose of this work is to understand how cellulose and starch as two biopolymers with similar structures interact in ZnCl_2_ solutions and how these interactions could influence the structure and properties of the resulting cellulose-starch hybrid materials. We first investigated the rheological properties of starch/cellulose/ZnCl_2_ solutions to discuss the molecular conformation between starch and cellulose. Then, we prepared cellulose-starch hybrid films with varied starch/cellulose ratios plasticized by an aqueous ZnCl_2_ solution. The structures of these films were investigated by scanning electron microscopy (SEM), X-ray diffraction (XRD), and Fourier-transform infrared (FTIR), and their mechanical properties were characterized.

## 2. Results and Discussion

### 2.1. Rheological Study of Cellulose/Starch/ZnCl_2_ Solutions

**Figure 1:**
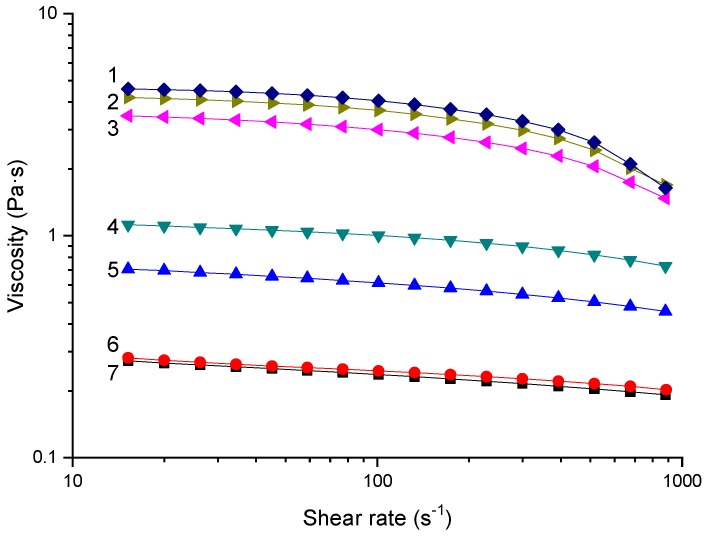
Viscosity-shear rate curves for 5 wt % solutes in 65 wt % ZnCl_2_ solution at 55 °C. Curve 1–7 represent different cellulose contents of 100 wt % (pure cellulose), 90 wt %, 70 wt %, 50 wt %, 30 wt %, 10 wt %, and 0 wt % (pure starch).

The rheological properties of cellulose/starch/ZnCl_2_ were characterized to investigate the interactions between biopolymer chains in ZnCl_2_ solutions. It can be seen from Figure 1 that starch/cellulose/ZnCl_2_ solutions at 55 °C were all shear-thinning fluids, which is in agreement with previous studies [78,87,88]. With reduced cellulose content in the solution, the viscosity decreased, which means that the contribution of the starch chains on the viscosity was less strong than that of the cellulose chains. In a previous study, the viscosity of cellulose/starch/ZnCl_2_ solutions increased with higher amylopectin contents [74]. Considering that a high-amylose (80%) starch was used here and amylose has a much lower molecular weight than amylopectin, it is likely that amylose is not as effective as amylopectin in contributing to the viscosity of the biopolymer solution. This is in agreement with our previous study, which showed that when dissolved in the same ZnCl_2_ solutions, the solution viscosity of the same high-amylose maize starch was lower than that of waxy maize starch (mostly amylopectin).

When the cellulose content was varied from 100 wt % to 70 wt %, curves 1, 2, and 3 were still quite close to each other. In contrast, curves 7, 6, and 5 deviated more when the cellulose content was changed from 0 wt % to 30 wt % (starch content from 100 wt % to 70 wt %). Moreover, for the samples with ≥70% cellulose content (curves 1, 2, and 3), their viscosity decreased sharply when the shear rate was higher than 500 s^−1^, indicating a stronger shear-thinning behavior. However, this drastic behavior could not be observed for the samples with lower cellulose contents. Because shear thinning is caused by the disentanglement of macromolecules under shear, it could be proposed that the entanglements between the cellulose chains were stronger than those between the starch (mostly amylose) chains.

In order to discuss the interactions between the amylose chains and cellulose chains, a mixing rule is introduced for the zero-shear-rate viscosity. Because the polymer concentration in the mixture is above the overlap concentration of each polymer, the log-additive model for the zero-shear-rate viscosity versus the cellulose content is used [74]:

(1) $$\ln\eta_{mix}=\phi_{1}\ln\eta_{1}+\phi_{2}\ln\eta_{2}.$$

In Equation (1), *η*_mix_ is the calculated viscosity; *ϕ*_1_ and *ϕ*_2_ are the dry weight fractions of cellulose and starch, respectively, with *ϕ*_1_ + *ϕ*_2_ = 1; and *η*_1_ and *η*_2_ are the viscosities of each component at *ϕ*_1_ = 1 and *ϕ*_2_ = 1, respectively. If the experimental values of viscosity are lower than the calculated ones (ln *η*_mix_), a new “compact” conformation between the polymers (e.g., interpolymer complexes) is formed. Oppositely, if the experimental viscosities are higher than the calculated ones, the components make “loose gel-like” or “branched” structures with looped and dangling ends [89,90].

**Figure 2:**
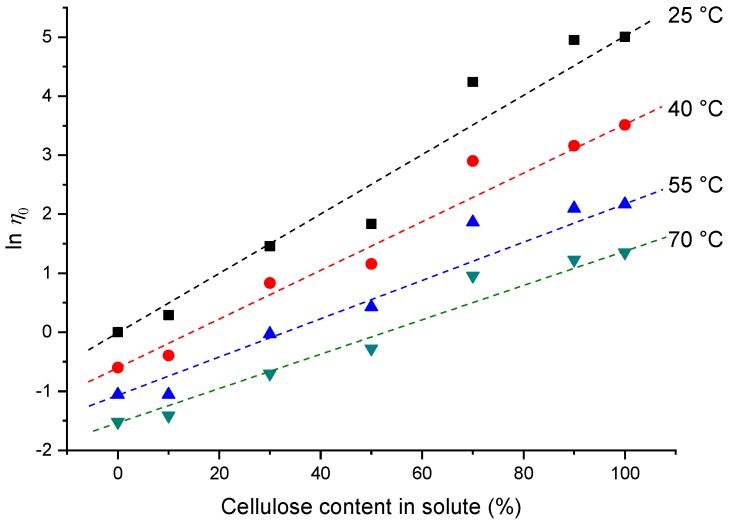
Zero-shear-rate viscosity versus cellulose content at different temperatures. The concentration of ZnCl_2_ solution was 65 wt %, and the total content of starch/cellulose was 5 wt %. The symbols represent experimental data, and the dashed lines were calculated according to Equation (1).

Figure 2 shows a comparison between the experimental zero-shear-rate viscosities and the calculated ones. Firstly, the zero-shear-rate viscosity increased with higher cellulose contents at different testing temperatures. Furthermore, when the cellulose content was ≤50 wt %, the experimental values were located below the dash lines. Because the dash lines are calculated according to Equation (1), it can be deduced that, for the samples with cellulose contents lower than 70 wt %, the interactions between the starch and the cellulose led to compact complexes. When the cellulose content was ≥70 wt %, the experimental values were located above the dash lines, indicating a loose or phase-separated structure. These interactions may affect the properties of the cellulose-starch hybrid materials, which will be discussed in Section 3.3.

Arrhenius activation energy can be obtained from the zero-shear-rate viscosity based on Equation (2):

(2) $$\ln\eta_{0}=\frac{E_{a}}{R\times T}.$$

**Figure 3:**
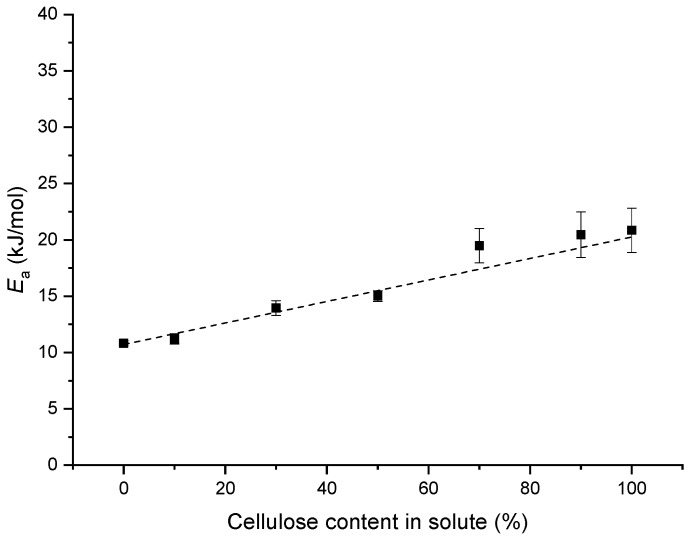
Activation energy (*E_a_*) of the starch/cellulose/ZnCl_2_ solutions as a function of cellulose content in the solute. The total solute is 5 wt %, and the dashed line is a linear fit. Error bars represent standard deviations.

In Equation (2), *T* is the Calvin temperature in K; *R* is the universal gas constant; and *E_a_* is the flow activation energy in kJ/mol [78,91,92]. Based on this equation, within the experimental error, *E_a_* increased linearly with higher cellulose contents in the solute (Figure 3). This reflects that the flowability or mobility of the cellulose chains in the ZnCl_2_ solutions was weaker than that of the starch (mostly amylose) chains. This is also the case for amylopectin/cellulose/[Emim][OAc] solutions, as reported previously [74], although *E_a_* for our starch/cellulose/ZnCl_2_ solution was much lower than that for an amylopectin/cellulose/[Emim][OAc] mixture. 

### 2.2. Structural Characterization of Cellulose-starch Hybrid Films

In this work, we used suitable methods to process cellulose-starch hybrid films of different formulations. When the cellulose content was ≥50 wt %, the films could only be prepared by solution casting with a ZnCl_2_ solution of 65 wt % concentration. However, when the cellulose content was ≤15 wt %, solution casting became unsuitable for preparing the associated films. In this case, the high acidity of the 65-wt % ZnCl_2_ solution (pH = 0.67) might cause serious acid hydrolysis of the starch macromolecules. Instead, the films could be prepared by compression molding using a 25-wt % ZnCl_2_ solution [61]. Nonetheless, the compression molding method only allows for formulations with a cellulose content of ≤15 wt %, otherwise films with good integrity could not be successfully formed due to the non-thermoplastic nature of cellulose.

#### 2.2.1. Morphology

**Figure 4:**
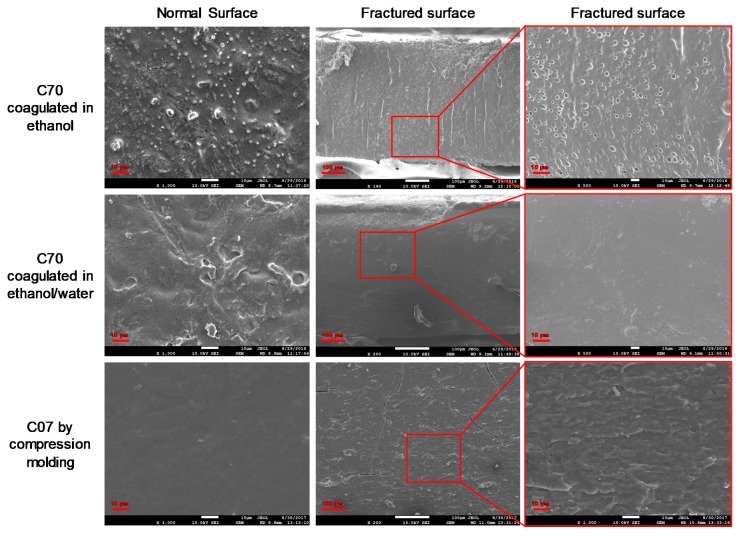
SEM images of cellulose-starch blend films. The magnifications are 1000 for normal-surface images and 200 and 500 for fractured-surface images.

When the solution casting was used, the hybrid materials were coagulated using either water or ethanol [74]. It can be seen from Figure 4 that for C70 hybrid films coagulated in ethanol, the normal surface was coarse and granular, while its fractured surface contained densely populated small holes. However, if an ethanol/water mixture solution was used for the coagulation of C70, a smoother and more homogeneous structure could be achieved. 

Ethanol has a strong dehydration effect on cellulose and starch, which may cause entanglement and coagulation of biopolymer chains to form films. We observed that with decreased cellulose content, more time was needed to form a film. This indicates that the entanglement and coagulation of the cellulose chains were easier than those of the starch chains. We propose that pure ethanol may lead to a difference in the coagulation rate between starch and cellulose, which may cause phase separation. However, an ethanol/water mixture solution could allow for the coagulation of both cellulose and starch in a gradual way; thus, phase separation could be restrained, and the inner structure of the hybrid films was more homogeneous. Therefore, we used ethanol/water mixture solutions as coagulation agents to prepare solution-casted biopolymer films with ≥50 wt % cellulose content in the following discussion. 

Figure 4 also shows the normal and fractured surfaces of C07 hybrid films prepared by hot pressing. The inner structure was observed to be homogeneous. We consider that a 65-wt % ZnCl_2_ solution could effectively promote the phase transition of native Gelose 80 starch and MCC.

#### 2.2.2. XRD Analysis

**Figure 5:**
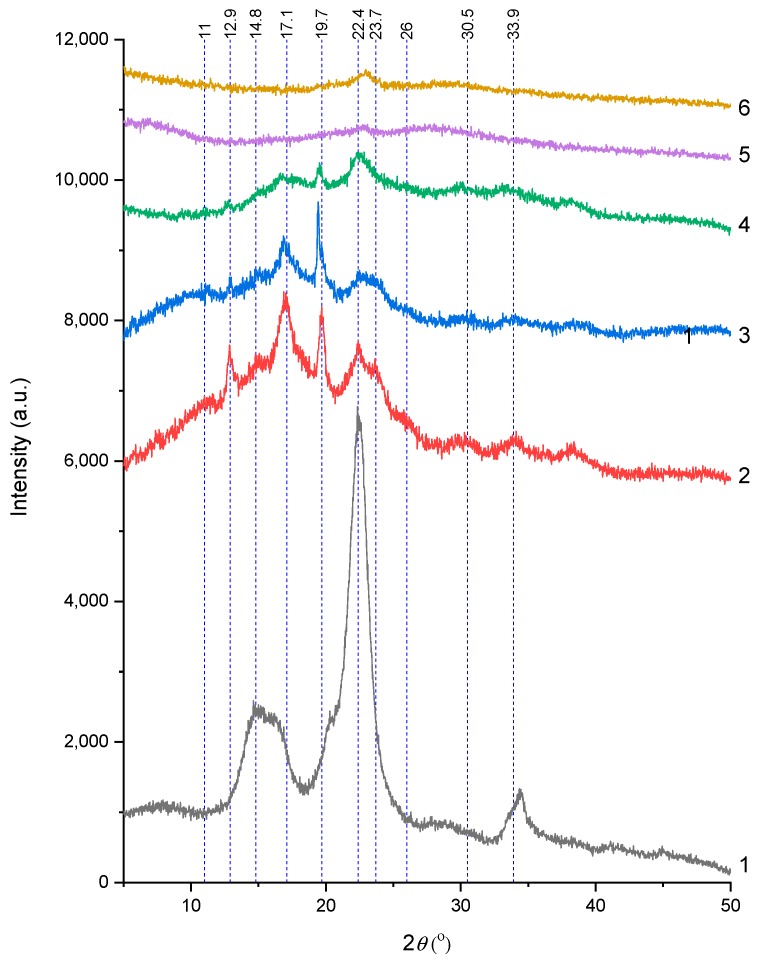
XRD results of (1) native microcrystalline cellulose (MCC), (2) native Gelose 80 starch, (3) C00 film, (4) C07 film, (5) C70 film, and (6) C100 film.

Figure 5 compares the XRD results of native cellulose, native Gelose 80 starch, and different biopolymer films. Native MCC powder (curve 1) shows classical diffraction peaks at 14.8° (plane 110, *d*-spacing = 0.60 nm) and 22.4° (plane 200, *d*-spacing = 0.40 nm), which are characteristic of the cellulose I structure [67,93,94]. Native Gelose 80 starch (curve 2) showed a strong diffraction peak at a 2*θ* position of around 17.1°, with a few smaller peaks at 2*θ* of 11°, 12.9°, 15°, 17.1°, 19.7°, 22.4°, 23.7°, 26°, 30.5°, and 33.9°, indicative of the B-type crystalline structure [95,96,97].

For the solution-casted C70 (curve 5) and C100 (curve 6) films, the original characteristic peaks of cellulose and starch nearly all vanished, except for the weak 22.6° peak. This indicates that the 65-wt % ZnCl_2_ solution caused a complete phase transition of MCC and the native Gelose 80 starch and that there was hardly any recrystallization of the starch or cellulose chains during preparation and storage. Previous studies have shown that starch tends to recrystallize during processing and storage [98,99,100]. Regenerated cellulose after dissolution in NaOH/thiourea aqueous solution or ionic liquids was reported to present the cellulose II structure [67,93,94]. Here, we consider that the reorganization and recrystallization of starch and cellulose might have been restricted by the ZnCl_2_ solution and cellulose chains.

For the compression-molded starch-based film C00 compared with the native Gelose 80 starch, some diffraction peaks disappeared, and the intensities of the peaks at 12.9° (very weak), 17.1°, 19.7°, and 22.4° reduced greatly. This suggests that the 25-wt % ZnCl_2_ solution could promote the phase transition of native starch granules at 130 °C. Moreover, the peaks for C07 (curve 4) weakened further compared with those for C00 (curve 3). In particular, the peak at 19.5° was suppressed most with the addition of cellulose. Again, cellulose might be able to limit the reorganization and recrystallization of starch chains.

#### 2.2.3. FTIR Analysis

**Figure 6:**
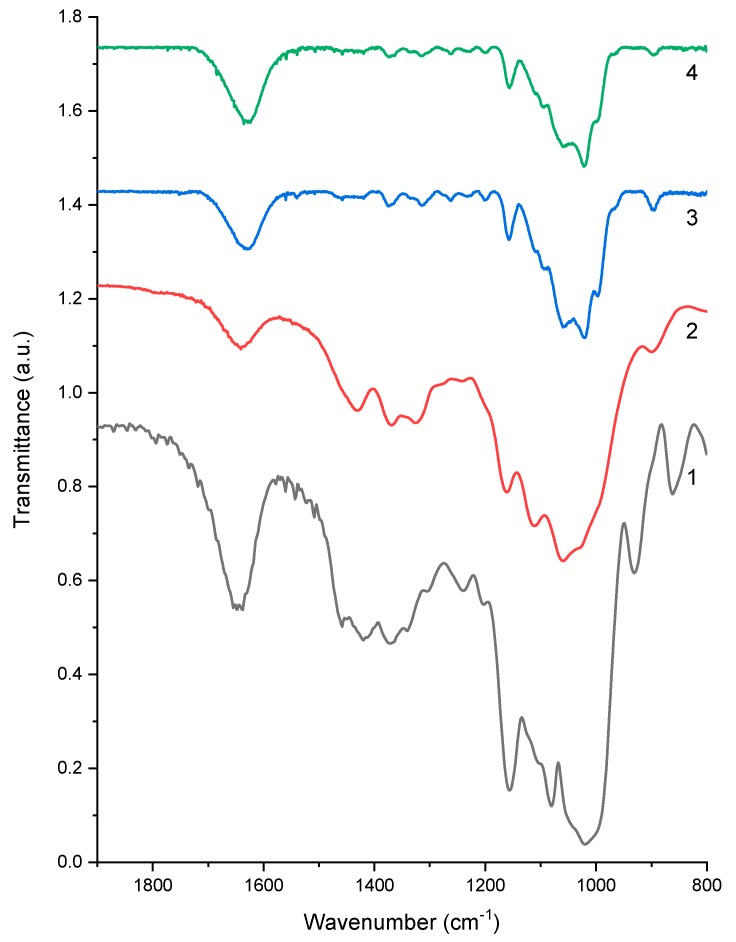
FTIR results of the (1) native Gelose 80 starch, (2) native MCC, (3) pure-cellulose film C100, and (4) cellulose-based blend film C70.

Figure 6 shows the FTIR spectra of the native MCC and biopolymer films. Native MCC powder (spectrum 1) shows FTIR bands similar to those reported previously [101,102,103]. Specifically, the band between 1640–1650 cm^−1^ represents the C=O vibration of hemiacetals; the band between 1432–1420 cm^−1^ reflects the CH_2_ scissoring vibration and also indicates the split of intramolecular hydrogen bonds concerning O at C6; the band between 1380–1350 cm^−1^ can be linked to the C–H bending vibration; the band between 1170–1158 cm^−1^ represents the C–O–C asymmetrical stretching vibration; and the band between 1112–1069 cm^−1^ suggests the C–OH skeletal vibration.

Compared with the native MCC, the pure-cellulose film C100 (spectrum 2) retained the most original FTIR peaks, except for the 1436 cm^−1^ peak, which vanished. Because this latter peak indicates intramolecular hydrogen bonding concerning O at C6 [74], its disappearance might suggest the disruption of the original structure of the MCC as a result of dissolution in the ZnCl_2_ solution and coagulation, which is in agreement with the XRD results. Moreover, there is no apparent difference in the FTIR bands between C70 (spectrum 3) and C100 (spectrum 2). This could be due to the same glycosidic unit of starch and cellulose. Moreover, it was likely that there were no new strong bonds formed between amylose and cellulose in the hybrid biopolymer materials, as suggested previously [74]. However, it is worth using other more sensitive techniques to understand the interactions (such as hydrogen bonding) between starch and cellulose. 

### 2.3. Mechanical Properties of Blend Films Plasticized by ZnCl_2_ Solution

**Figure 7:**
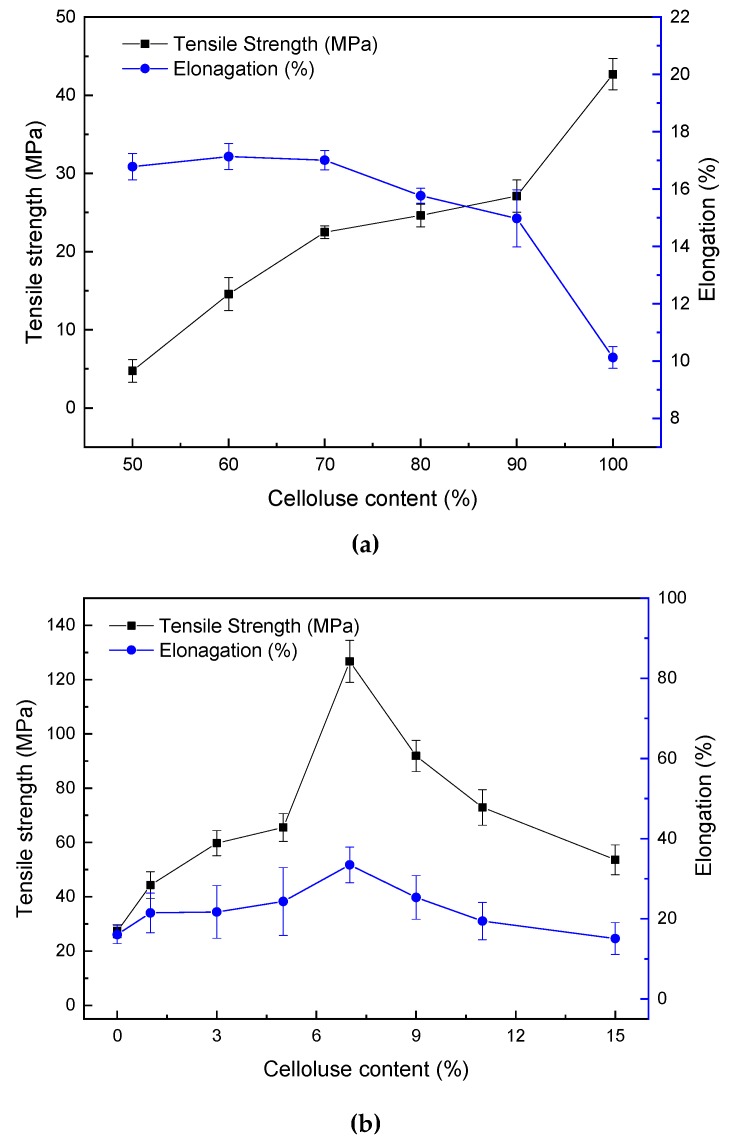
The mechanical properties for starch/cellulose blend materials with different cellulose content by (**a**) a casting method and(**b**) a compression molding method. Error bars represent standard deviations.

Figure 7 shows the mechanical properties of cellulose-rich films prepared by solution casting with a 65-wt % ZnCl_2_ solution and starch-rich films prepared by compression molding with a 25-wt % ZnCl_2_ solution. Figure 7a shows that the pure-cellulose film C100 had *σ*_t_ of 42.7 ± 2.0 MPa and *ε*_b_ of 10.1 ± 0.4%. In a previous study, regenerated cellulose films from [Amim][Cl] had a DP of 480 and *σ*_t_ of 138 MPa [67]. Our lower value here could be due to the acid hydrolysis of the biopolymers at a high pH with a 65-wt % ZnCl_2_ solution. For cellulose-rich hybrid films, the increased starch content led to higher *ε*_b_ but lower *σ*_t_. The reason could be that starch may have partly destroyed the intermolecular interactions of cellulose, while the latter was mainly responsible for the mechanical properties. Moreover, as starch and cellulose were phase-separated in the blends (as indicated by the rheological results), a higher content of starch could have destroyed the continuous phase of cellulose, which may have also led to reduced mechanical properties. 

Figure 7b shows that *σ*_t_ of the pure-starch film C00 was close to the values reported in our previous study [79]. With the addition of cellulose into starch, both *σ*_t_ and *ε*_b_ of the resultant hybrid films increased initially and then decreased. Both the highest *σ*_t_ (126.7 ± 7.8 MPa) and *ε*_b_ (33.5 ± 4.4%) was achieved at a 7-wt % cellulose content. This suggests that a small amount (7 wt %) of cellulose in starch could provide a reinforcement effect on the hybrid films, although C07 had a lower degree of crystallinity (as shown by XRD results). This may be ascribed to the hydrogen bonding interactions between starch and cellulose due to their similar chemical structures and the inherently better mechanical properties of cellulose. Unfortunately, pure-cellulose films and pure-starch films were unable to be prepared using the same method, so a comparison of the mechanical properties between the two types of biopolymer film was not possible. Moreover, the rheological study shows that for starch-rich materials, interactions between starch (mostly amylose) and cellulose resulted in new compact complexes, which might reinforce the blend films. However, too much cellulose in a starch matrix may lead to phase separation and, thus, deteriorated mechanical properties. 

It can be seen that both *σ*_t_ and *ε*_b_ of the starch-rich hybrid films prepared by compression molding (Figure 7b) were much higher than those of the cellulose-rich hybrid films prepared by solution casting (Figure 7a). Because a 65-wt % ZnCl_2_ solution was used for solution casting, which is rather acidic (pH = 0.67), it was likely to cause acid hydrolysis of both the cellulose and starch during the solution casting, which could weaken the mechanical properties of the films. 

## 3. Materials and Methods

### 3.1. Materials

Gelose 80 maize starch (about 80% amylose content, as determined by the manufacturer) was supplied by National Starch Pty Ltd. (Lane Cove, Australia). α-Microcrystalline cellulose (MCC; Product No. C804602, CAS: 9004-34-6) was supplied by Shanghai Macklin Biochemical Co., Ltd. (Shanghai, China). Anhydrous zinc chloride (ZnCl_2_) and ethanol of analytical grade were purchased from Guangzhou Chemical Reagent Factory (Guangzhou, China). All the solutions were prepared with distilled water.

### 3.2. Materials Preparation

Table 1 shows the sample formulations of the different cellulose-starch materials. First, ZnCl_2_ aqueous solutions of two concentrations (65 and 25 wt %) were prepared by mixing ZnCl_2_ and H_2_O at ratios of 65/35 and 25/75 (wt./wt.).

Cellulose-starch hybrid materials with a ratio of cellulose of higher than 50 wt % were prepared via a dissolution-casting-coagulation route [74]. First, certain amounts of Gelose 80 starch or MCC were added into a ZnCl_2_ aqueous solution of 65 wt % concentration in a sealed reaction vessel followed by constant stirring at 50 °C for 1.5 h to allow for complete dissolution. Then, about 30 g of the solution was poured onto a petri dish and spread evenly. The biopolymer was then coagulated in two ways: (1) using absolute ethanol for 24 h; and (2) using ethanol solutions with concentrations of 30 wt %, 50 wt %, and 70 wt %, with each concentration kept for 1 h, followed by treatment with absolute ethanol at room temperature for another 21 h. The bath volume was 10 times higher than the solution volume, and the obtained wet films were dried in a blast-drying oven at 50 °C for 1 h. The thickness of the materials was about 0.4–0.5 mm. Our preliminary work showed that ZnCl_2_ solutions of lower concentrations than 65 wt % cannot dissolve MCC completely.

Cellulose-starch hybrid materials with a ratio of cellulose of less than 15 wt % were prepared by a compression molding method [60,61,62,66]. Specifically, starch and MCC powders were mixed using a blender, with the addition of a ZnCl_2_ solution of 25 wt % concentration. Our previous study showed that this concentration of ZnCl_2_ can provide a reinforcement effect on starch-based materials [79]. Then, the blended powder (25 g in total) was equally spread over the molding area (15 cm × 15 cm = 225 cm^2^) with poly(tetrafluoroethylene) (PTFE) glass fabrics located between the starch and the mold. For the subsequent compression molding, a flat sulfuration machine (Guangzhou Shunchuang Rubber Machinery Factory, Guangzhou, China) was used, which has two press areas with the upper one for hot press and the lower one maintained at room temperature for cooling. Each sample in the mold was first hot-pressed in the upper area at a temperature of 120 °C and a pressure of 10 MPa for 6 min and then immediately moved to the lower area and pressed under 10 MPa for cooling for 2 min. Subsequently, the mold was opened and the sample was retrieved (thickness about 1.2 mm). Our preliminary work showed that for compression molding, ZnCl_2_ solutions of higher concentrations than 25 wt % are not effective for proper plasticization of the biopolymer materials.

All the materials were conditioned at 75% relative humidity (with oversaturated sodium chloride solution) for 7 days before characterization of the materials. 

### 3.3. Characterization

#### 3.3.1. Rheological Properties

Rheological measurements of cellulose/starch/ZnCl_2_ solutions were carried out using an Anton Paar MCR 92 rheometer (Anton Paar GmbH, Graz, Austria) with a 60-mm-diameter plate geometry and a Peltier temperature control system. Silicone oil (DC 200, Sigma-Aldrich) was placed around the edge of the measuring cell to prevent the absorption of water from the environment. Silicone oil would hardly affect the experimental results, as it is immiscible with polysaccharide solutions and has a relatively low viscosity (9.5 mPa·s at 20 °C) [88,91]. Steady-shear viscosities of starch/cellulose/ZnCl_2_ solutions were recorded at shear rates from 10 to 1000 s^−1^ at certain temperatures of 25 °C, 40 °C, 55 °C, and 70 °C. The concentration of ZnCl_2_ solution was fixed at 65%, which can dissolve cellulose and starch totally, and the concentration of starch and cellulose was set at 5% (dry weight). The different starch/cellulose ratios tested were 0 wt % (pure cellulose), 10 wt %, 30 wt %, 50 wt %, 70 wt %, 90 wt %, and 100 wt % (pure starch). The rheological properties of a 65-wt % ZnCl_2_ solution were also detected. All the rheological tests were carried out at least twice to ensure the consistency of the results. 

#### 3.3.2. Scanning Electron Microscopy

The morphologies of the normal and fractured surfaces of the cellulose-starch films were examined using a scanning electron microscope (JEOL JSM-7001F, Tokyo, Japan) with an accelerating voltage of 10 kV and a spot size of 6 nm. The blend films were cryo-ground in liquid nitrogen to obtain fractured surfaces, and the pieces were then fixed onto circular metal stubs previously covered with double-sided adhesive, followed by platinum coating for a 5-nm thickness using an Eiko sputter coater under a vacuum.

#### 3.3.3. X-ray Diffraction (XRD)

X-ray diffraction (XRD) analysis was performed with an Xpert PRO diffractometer (PANalytical B.V., Almelo, The Netherlands) operated at 40 mA and 40 kV using Cu Kα radiation with a wavelength of 0.1542 nm as the X-ray source. The scanning was undertaken with the diffraction angle (2*θ*) from 5° to 50° with a scanning speed of 10 °/min and a scanning step of 0.033°.

#### 3.3.4. Fourier-Transform Infrared Spectroscopy

Fourier-transform infrared (FTIR) spectra for the native MCC powder, pure-cellulose films and hybrid films were obtained in the range of 1900–800 cm^−1^ using a TENSOR27 FTIR model manufactured by Burker, Germany. Native starch or MCC powder was mixed with KBr and well ground before being pressed into wafers. The films were detected using an attenuated total reflectance (ATR) accessory, which contained a ZnSe crystal at a nominal incident angle of 45°, yielding about 12 internal reflections at the sample surface. All the resolutions were 4 cm^−1^, and all the spectra were recorded at room temperature (25 °C).

#### 3.3.5. Mechanical Properties

The mechanical properties of the blend films were determined using an Instron 5566 Universal Testing Machine with a 500 N load cell (Instron (Shanghai) Limited, Shanghai, China). The films were cut into dumbbell-shaped strips, according to ASTM D882-10. The testing was performed with a constant deformation rate of 10 mm/min at room temperature. The tensile strength (*σ*_t_) and elongation at the break (*ε*_b_) were determined by Instron Merlin software version 2.3 (Instron (Shanghai) Limited, Shanghai, China) from at least 7 specimens for each sample [9].

## 4. Conclusions

In this work, we studied the molecular interactions between starch and cellulose and the properties of such hybrid films based on these two polysaccharides. For starch-rich hybrid films prepared by compression molding, a small amount (≤7 wt %) of cellulose was found to improve the mechanical properties despite the reduced crystallinity of starch; and for cellulose-rich hybrid films, a higher content of starch reduced the material properties although chemical interactions were not apparently influenced. One possible reason could be the inherently better mechanical properties of cellulose, notwithstanding that this could not be verified in this current work. Moreover, the rheological study of the cellulose/starch/ZnCl_2_ solutions indicated that with ≤50-wt % cellulose content, the interactions between the starch (mostly amylose) and the cellulose resulted in compact complexes (interfacial interactions), whereas with ≥70-wt % cellulose content, the interactions led to a loose structure (phase separation). These different ways of interactions and structural conformation could also account for the observed differences in the mechanical properties. Thus, our results allow for a further understanding of the molecular interactions between the starch and the cellulose as two polysaccharides with very similar chemical structures. The knowledge obtained from this work could provide insights into the future development of biodegradable materials based on these natural polymers with tailored structures and properties for food packaging and various biomedical (e.g., drug delivery) and environmental (e.g., absorption and controlled release) applications.

## Figures and Tables

**Table 1:** Sample formulations of the cellulose-starch hybrid films.

Samples	Method	Starch (g)	Cellulose (g)	Concentration of ZnCl_2_ Solution (wt %)	ZnCl_2_ Solution (g)
C00	CM	100.0	0.0	25	35
C01	CM	99.0	1.0	25	35
C03	CM	97.0	2.0	25	35
C05	CM	95.0	5.0	25	35
C07	CM	93.0	7.0	25	35
C09	CM	91.0	9.0	25	35
C11	CM	89.0	11.0	25	35
C13	CM	87.0	13.0	25	35
C15	CM	85.0	15.0	25	35
C50	SC	2.5	2.5	65	95
C60	SC	2.0	3.0	65	95
C70	SC	1.5	3.5	65	95
C80	SC	1.0	4.0	65	95
C90	SC	0.5	4.5	65	95
C100	SC	0.0	5.0	65	95

CM, compression molding; SC, solution casting.

## Abbreviations

MCC	Microcrystalline cellulose
SEM	scanning electron microscopy
XRD	X-ray diffraction
FTIR	Fourier-transform infrared
ATR	Attenuated Total Reflectance
[Amim][Cl]	1-allyl-3-methylimidazolium chloride
[Emin][OAc]	1-ethyl-3-methylimidazolium acetate
[Bmim][OAc]	1-butyl-3-methylimidazolium chloride
